# Analysis by *siRNA_profile *program displays novel thermodynamic characteristics of highly functional siRNA molecules

**DOI:** 10.1186/1751-0473-3-8

**Published:** 2008-05-21

**Authors:** Pirkko Muhonen, Ranga N Parthasarathy, Anthony J Janckila, Kalman G Büki, H Kalervo Väänänen

**Affiliations:** 1 Institute of Biomedicine, Department of Anatomy, University of Turku, Kiinamyllynkatu 10, FIN-20520 Turku, Finland; 2 Veterans Affairs Medical Center, and University of Louisville, Louisville, KY, USA

## Abstract

**Objective:**

Here we report the improved results of a new siRNA design program and analysis tool called ***siRNA_profile ***that reveals an additional criterion for bioinformatic search of highly functional siRNA sequences.

**Methods:**

We retrospectively analysed over 2400 siRNA sequences from 34 genes and with known efficacies to categorize factors that differentiate highly, moderately and non-functional siRNA sequences in more detail. We tested the biological relevance of ***siRNA_profile ***in CHO cells stably expressing human TRACP.

**Results:**

The highly functional siRNA molecules exhibited lower overall stabilities than non-functional siRNAs after taking into consideration all the nucleotides from 5'-terminus to the 3'-terminus along the siRNA molecule, in addition to the 5'-section of the antisense strand and the region between 9–14 nucleotides as previously has been acknowledged. Comparison of the ***siRNA_profile ***program to five other programs resulted in a wide range of selected siRNA sequences with diverse gene silencing capacities, even when the target was only 197 nucleotides long. Six siRNA design programs selected 24 different siRNA sequences, and only 6 of them were selected by two or more programs. The other 18 sequences were individually selected by these six programs.

**Conclusion:**

Low general stability of dsRNA plays a significant role in the RNAi pathway and is a recommended criterion to consider, in addition to 5'-instability, internal instability, nucleotide preferences and target mRNA position, when designing highly efficient siRNAs.

## Introduction

RNA interference (RNAi) is a gene silencing mechanism where short interfering RNA (siRNAs) and microRNA (miRNAs) molecules inhibit the transcription and translation of target genes in a sequence-specific manner [1-3]. siRNAs are exogenously produced ~21 nucleotides long, double stranded RNA molecules with complete complementarity to the target sequence. miRNAs are a family of endogenously encoded small non-coding RNAs, derived by processing of short RNA hairpins, that can inhibit the translation of mRNAs bearing partial complementarity to the target sequences. RNAi has been acknowledged as a practical tool for new drug target discovery and RNAi drug development in mammalian cells [4]. Therefore designing highly functional siRNA molecules has become an essential part of RNAi methodology. We have developed a novel and user-friendly siRNA design algorithm *siRNA_profile *with multiple options for minimizing the identification of non-functional, unspecific and immunostimulatory siRNA molecules. The analysis of functional and non-functional siRNA molecules were done by the *siRNA_profile *program to demonstrate in more detail the characteristics of highly functional siRNA molecules to help scientists in their search for theoretically and biologically efficient siRNAs.

### Conventional siRNA design criteria

The rapid development of RNAi applications has revealed the need for efficient and specific siRNA design and analysis tools to maximize the efficiency while minimizing possible side-effects [5]. Computational methods and neural networks are tools approaching ideal siRNA design; however, so far none of them are perfect. Currently, thermodynamic characteristics of functional siRNA molecules guide siRNA design strategy. First, it has been shown that thermodynamic differences in the base-pairing stabilities of the 5'-ends of both siRNA and miRNA molecules play a critical role in determining which strand initiates RNA induced silencing complex (RISC) activation [6,7]. To achieve an efficient RNAi effect, activated RISC should be able to silence multiple copies of the target mRNA. A second criterion for effective siRNAs is low internal stability in the cleavage region between 9 to 14 nucleotides (calculated from the 5'-terminus) of the antisense strand. This is believed to have a critical role in mRNA cleavage and it may also help to release RISC from the cleaved target [7]. Third, there are findings of the nucleotide preferences over the length of siRNA sequence [8,9].

### *siRNA_profile *program design

The *siRNA_profile *program and a full text including additional data and a printable help page are available in the *siRNA_profile *program web page [10]. Briefly, the *siRNA_profile *program is based on findings of the asymmetric differences between functional and non-functional siRNAs [6,7,11] and on our studies of positional nucleotide differences and average dsRNA stability along the siRNA antisense strand. We have also incorporated some recommendations of Elbashir, S.M., et al. [2] and developed a novel, interactive and user-friendly siRNA design algorithm with multiple options for minimizing unspecific siRNA design. Previously, it has been recognized that 5'-UGUGU-3' motifs have immunostimulatory potential in synthetic siRNA molecules, in addition to CpG motifs in single stranded RNAs and DNA oligomers [12-14]. We have incorporated a sensor able to recognize immunostimulatory motifs intending to avoid unnecessary false phenotypes by siRNA molecules and, additionally, by anti-miRNA oligomers. In addition, a scoring system was integrated into the *siRNA_profile *program. It was adjusted based on our findings on nucleotide, purine and pyrimidine distribution along functional and non-functional siRNA sequences [the scoring system described in *siRNA_profile *web page].

The *siRNA_profile *program uses free energy values for calculation of average internal stability profiles. The average internal stability profiles were calculated as the sum of stability in five nucleotide windows by using the nearest-neighbour method as previously described [7,11]. Our program browses and calculates the target sequence from the antisense point of view, from the 5' - to the 3'- direction. However, it does not utilize nucleotides on the mRNA beyond the 3'- end of the siRNA as previously has been described [7], because this calculation method may easily lead to false bending of the profile. This unique and beneficial feature of the *siRNA_profile *program allows the calculation of the factual, not the false dsRNA thermodynamic characters.

The *siRNA_profile *program was originally developed on Red Hat Linux 9.0 and compiled to an executable file. The algorithm was written in C-programming language and the CGI application was written in Perl programming language. The application uses the user-given input values from the *siRNA_profile *website to analyze and display the results.

## Findings

**Figure 1:**
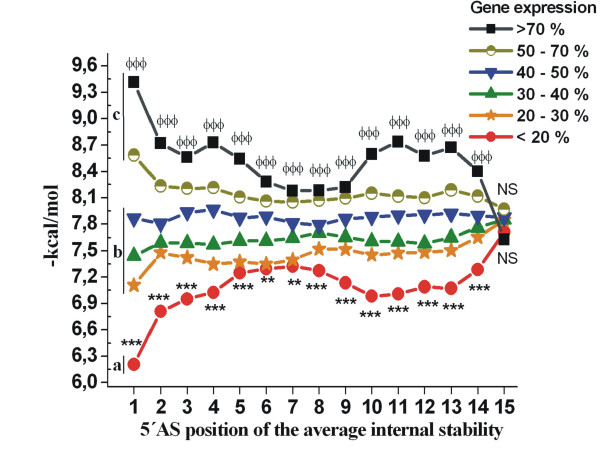
**siRNA stability analysis by *siRNA_profile *and novel guidelines for highly efficient siRNA design**. The average energy profiles of 2431 siRNA sequences [15] were calculated and analyzed by ***siRNA_profile ***program. The average energy profiles of siRNA sequences were divided into categories according to their efficiencies (GE categories: ≤ 20%, 20.1%–30%, 30.1%–40%, 40.1%–50%, 50.1%–70% and ≤70%). The siRNA efficiency showed siRNA instability dependency: highly functional siRNA molecules (GE ≤ 20%, group a) illustrated lower overall stability in addition to asymmetric average energy profile than moderately (GE 20.1 – 50%, group b) or non-functional (GE >50%, group c) siRNA molecules. Statistical analyses were performed with one-way ANOVA: (*) average internal stabilities of highly functional siRNAs (a) compared to moderately functional ones (b); Φ average internal stabilities of moderately functional siRNAs (b) compared to non-functional ones (c). ** p < 0.01; ***/ΦΦΦ p < 0.001.

In order to understand the similarities in biologically effective siRNA sequences, previously published siRNA data were used [15]. 2431 siRNA sequences from 34 genes were fed into the *siRNA_profile *program and siRNA profiles were divided into categories based on gene expression levels after siRNA silencing (gene expression level (GE) categories: ≤ 20%, n = 280; 20.1%–30%, n = 455; 30.1%–40%, n = 445; 40.1%–50%, n = 405; 50.1%–70%, n = 736 and >70%, n = 110). The functional and non-functional siRNA sequences exhibited "the mirror energy profiles" (Figure 1). Low stability in the 5'-end of the antisense strand was a critical factor in determining the correct strand activating RISC and further catalysing mRNA degradation. This asymmetry property is in good agreement with previous results [6,7]. In addition, our results showed that highly functional siRNAs exhibited lower overall internal stabilities than non-functional siRNAs (Figure 1). The low overall stability properties of the most effective siRNAs were apparent at all the nucleotides from 5'-terminus to the 3'-terminus along the siRNA molecule excluding the last one. Noticeably, there was no position along the length of functional and non-functional siRNAs at which energy profiles crossed. This was true for both 5'-region and the region from nucleotides 9–14. These novel features were found to correlate siRNA energy profiles with their efficiencies. To demonstrate the differences of average internal stabilities of highly functional, moderately functional and non-functional siRNAs in more detail, these siRNAs were compared to each other. Average internal stability of highly functional siRNAs (Figure 1, GE <20%, marked as group a) showed significantly lower internal stability compared to moderately functional siRNAs (GE 20.1–50%, marked as group b). Non-functional siRNAs (GE 50–100%, marked as group c) exhibited significantly higher average internal stability than moderately functional ones. Here, our results obtained by *siRNA_profile *program suggest that the thermodynamic characteristics focusing only on the 5'-end stability properties of the antisense strand are not enough to differentiate highly functional siRNAs from the moderately functional or non-functional ones.

To demonstrate the usefulness of *siRNA_profile *program as a siRNA candidate search service, the biological efficacies of siRNA molecules targeting tartrate-resistant acid phosphatase (TRACP) experimentally designed by *siRNA_ profile *were validated in a Chinese Hamster Ovary (CHO) cell line stably over-expressing human TRACP under a CMV promoter [16] [data shown in *siRNA_profile *web page].

The **siRNA_profile **program functionality was compared to five other available siRNA design programs. Human CyclophilinB sequence region 193 – 390 [GenBank:M60857] [8] was fed into the ***siRNA_profile ***program, Deqor [17], siRNA target finder (Ambion, USA), siDesign Center (Dharmacon, USA), EMBOSS explorer (gwilliam ^© ^rfcgr.mrc.ac.uk) and siDirect [18], and optimal siRNA candidate search options were used. siRNA design results were compared to results obtained by ***siRNA_profile ***by using the known efficacies of each siRNA sequences targeting the selected human Cyclophilin region (Table 1). The results showed that all tested programs selected functional siRNA sequences with significant variability. The novel ***siRNA_profile ***program showed the highest selectivity. However, we noticed that all six programs tend to select different siRNA sequences even though the mRNA region selected for candidate search was only 197 nucleotides long. Programs selected altogether 24 different sequences, and only 6 of them were selected by two or more programs. The other 18 sequences were individually selected by these six programs. This result shows the dilemma, that even for programs that operate with related selection parameters, very different sequences were chosen with a range of siRNA efficacies.

**Table 1:** Comparison of the *siRNA_profile *program and five other siRNA design programs

	***siRNA_profile***	**Deqor**	**Ambion**	**siDesign**	**EMBOSS**	**SiDirect**
**Number of hits**	6 (4)	4(1)	3(0)	2(2)	6(4)	9(7)
Average GE%*	10.4	14.8	21.1	13.0	21.6	14.8
Median%*	10.2	13.1	26.9	13.0	16.1	14.9
Minimum GE%*	5.4	10.3	8.1	12.6	6.7	5.8
Maximum GE%*	15.1	22.8	28.3	13.3	54.1	32.2

197 nt human CyclophilinB sequence and data of known siRNA efficacies [8] were used for siRNA design program comparison. Number of hits that were not selected by any of five other programs is shown in brackets (). * Gene expression (GE) % of the baseline

In conclusion, our results suggest that low general stability of dsRNA plays a significant role in the RNAi pathway and is recommended to be used as an additional guideline for highly efficient siRNA design. Additionally, all known immunostimulatory motifs to date are highlighted in colour to prevent analysis of false phenotypes and the output of the *siRNA_profile *program displays the average internal energy profiles for each siRNA candidate. According to our results, the siRNA sequences exhibiting both favourable asymmetric properties *and *low overall stabilities according to Figure 1 are recommended for gene knock down experiments.

## Availability and requirements

***siRNA_profile *program availability**: 

## Competing interests

The authors declare that they have no competing interests.

## Authors' contributions

PM, KGB, HKV Study concept and design. PM, KGB Acquisition of the data. PM Analysis and interpretation of the data. AJJ, RNP CHO cell line stably expressing human tartrate-resistant acid phosphatase [additional data in the *siRNA_profile *web page]. PM Drafting of the manuscript. HKV Study supervision. All authors read and approved the final manuscript.
